# Supplementary material for: Perturbed N-glycosylation of Halobacterium salinarum archaellum filaments leads to filament bundling and compromised cell motility
Source: Nat Commun. 2024 Jul 11;15:5841. doi: 10.1038/s41467-024-50277-1 (PMC11239922; doi:10.1038/s41467-024-50277-1)
Supplement: Supplementary file 1 — Supplementary Information [file 41467_2024_50277_MOESM1_ESM.pdf]

Supplementary Information for:

Perturbed N-glycosylation of *Halobacterium salinarum* archaellum filaments leads to filament bundling and compromised cell motility

Shahar Sofer[1,#], Zlata Vershinin[1,#], Leen Mashni[1,#], Ran Zalk[2], Anat Shahar[2], Jerry Eichler[1] & Iris Grossman-Haham[1,2,*]

[1]Department of Life Sciences, Ben-Gurion University of the Negev, Beer Sheva, Israel

[2]The Ilse Katz Institute for Nanoscale Science and Technology, Ben-Gurion University of the Negev, Beer Sheva, Israel

[#]These authors contributed equally to this work

*Correspondence should be sent to: Iris Grossman-Haham, Dept. of Life Sciences, Ben-Gurion University of the Negev, P.O. Box 653, Beersheva 84105, Israel. Tel: 972 8646 1368; email: irisgh@bgu.ac.il

Supplementary Information includes:

Supplementary Figures 1-10

Supplementary Tables 1-3

Supplementary References

Supplementary Figures

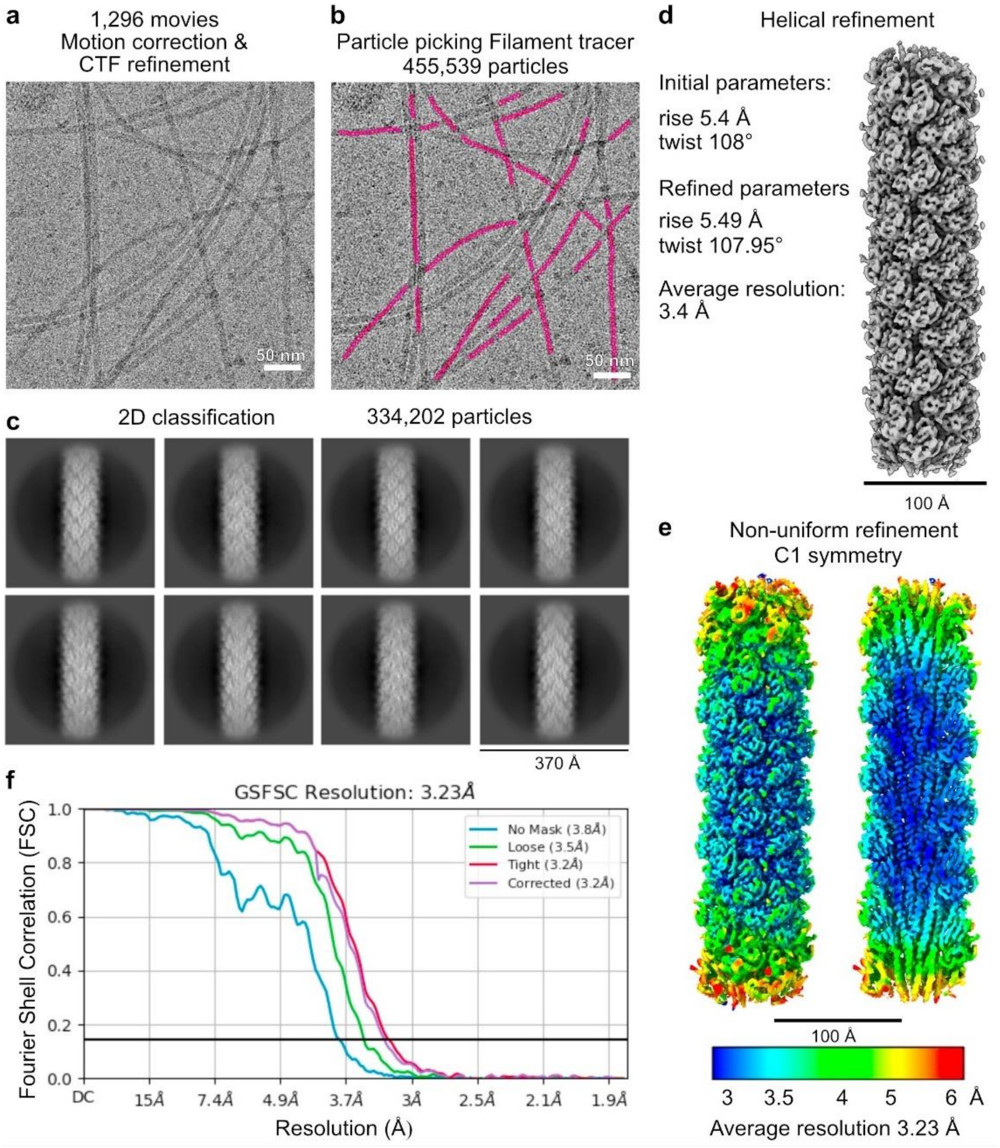

Supplementary Fig. 1. ***Hbt. salinarum*** **archaellum filament cryo-EM data processing workflow. a.** Representative cryo-EM micrograph. **b.** Examples of particles picked from the micrograph shown in **a** using Filament tracer in cryoSPARC[1]. **c.** Two-dimensional class averages selected for further processing (out of 50). **d.** Cryo-EM map of the *Hbt. salinarum* archaellum filament obtained by applying helical symmetry (initial and refined parameters are indicated). **e.** Cryo-EM map of the *Hbt. salinarum* archaellum filament obtained without applying helical symmetry. The map is colored according to local resolution, as estimated by CryoSPARC[1]. Left – surface view. Right – cross-section parallel to the long axis of the filament. **f.** Fourier Shell Coefficient (FSC) measured by the Gold-standard method.

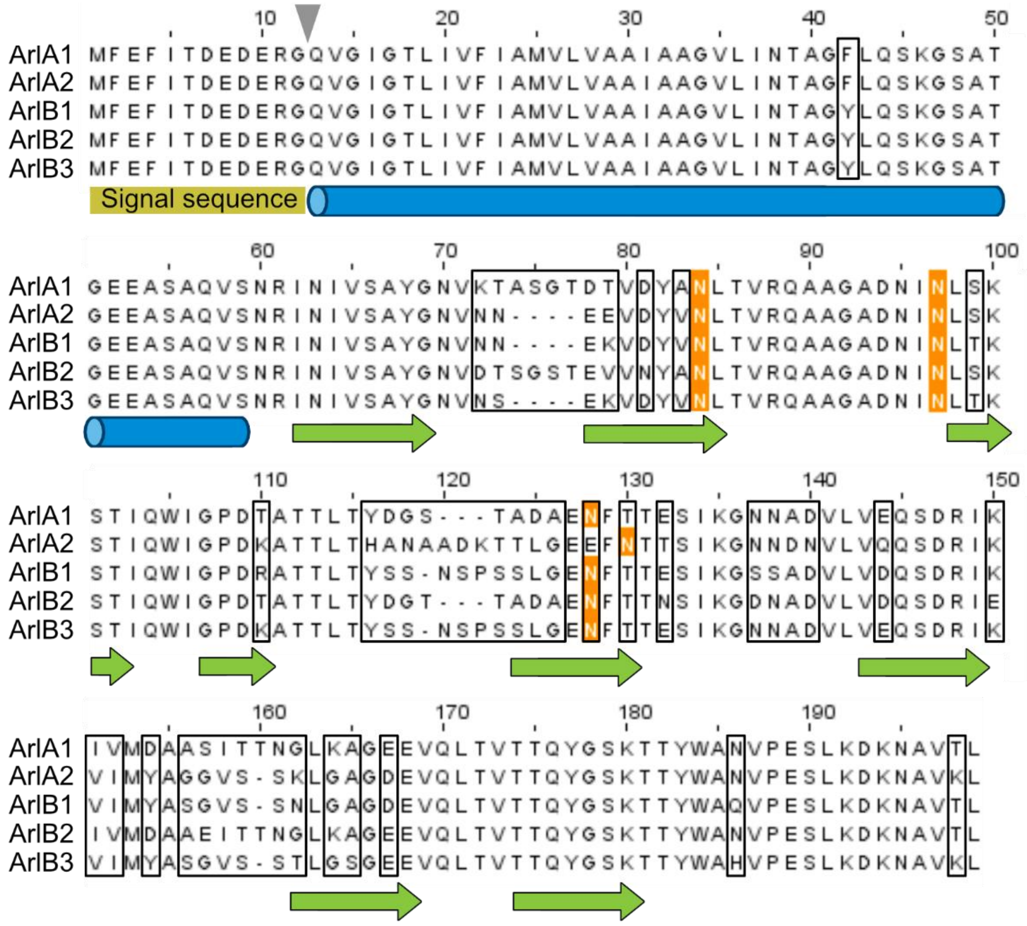

Supplementary Fig. 2. Comparison of *Hbt. salinarum* archaellin sequences. Multiple-sequence alignment of the five *Hbt. salinarum* archaellins was generated using ClustalΩ[2,3]. A grey arrowhead marks the position of signal sequence cleavage, according to the density seen in the cryo-EM map. The same cleavage site was predicted using the FlaFind server[4]. Blue cylinders indicate α-helices and green arrows indicate β-strands. Black boxes mark positions in the sequences that vary among archaellins. Most of the variable regions do not have a defined secondary structure. Asparagine residues known to be glycosylated[3] are indicated against an orange background.

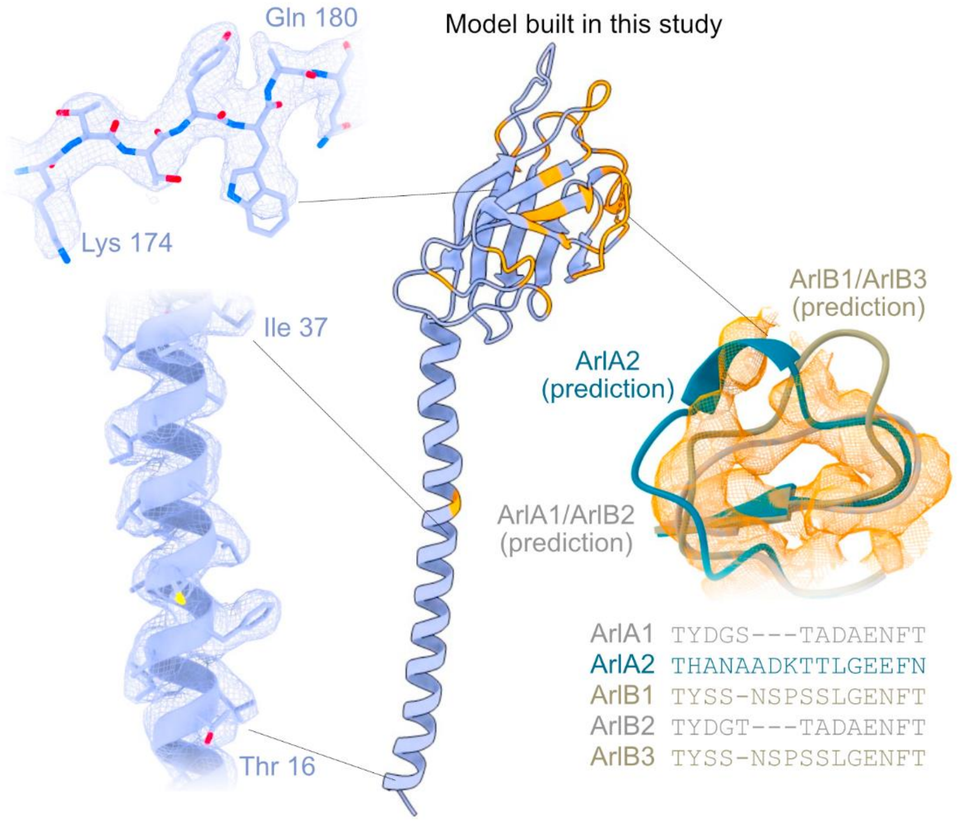

Supplementary Fig. 3. Quality of the cryo-EM map. Cartoon presentation of a model of one archaellin (center), in which residues conserved among *Hbt. salinarum* archaellins are colored purple and residues that vary are colored orange. Left (purple) – two examples of the cryo-EM map of conserved regions, where clear side-chain density is observed. Right (orange) – example of a poor-quality region in the cryo-EM map of a sequence that differs among archaellins (indicated by the sequence alignment). Three AlphaFold2 structure predictions[5] for *Hbt. salinarum* archaellins are shown (the predictions for ArlB1 and ArlB3 in this region are essentially the same, and thus only one is displayed; the same is true for ArlA1 and ArlB2), being placed into the density by alignment to the model of conserved regions. None of the predictions seems to fit well into the map, such that the identity of the archaellin could not be determined. Consequently, alanine residues were modelled into this region (Supplementary Table 2). See Supplementary Fig. 4 for more detail.

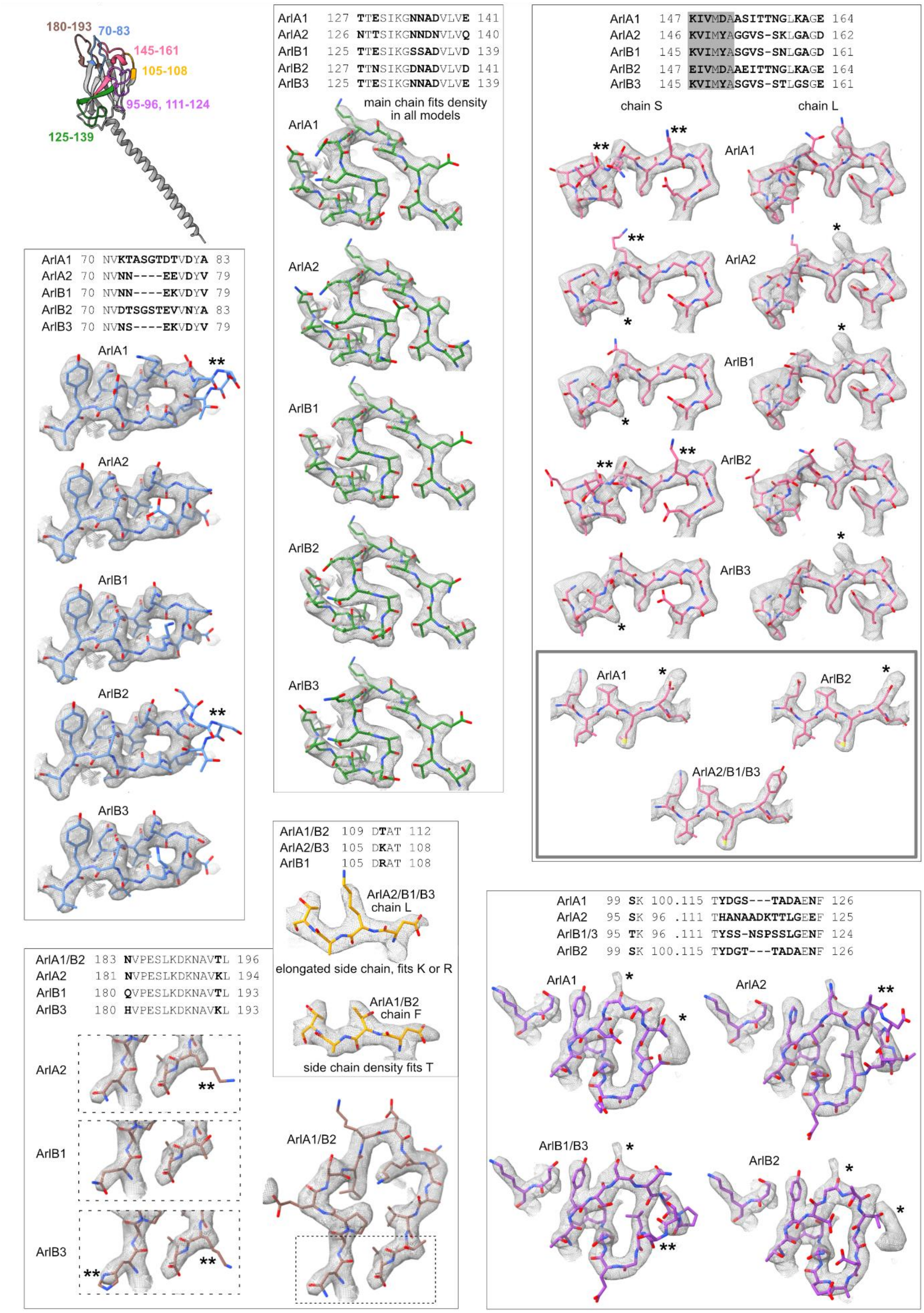

Supplementary Fig. 4. Cryo-EM densities in regions that differ among archaellins. Top left – model of one archaellin with colored regions differing across archaellins (numbering according to ArlB1). Each box focuses on one of those regions, displaying sequence alignment

of the five *Hbt. salinarum* archaellins, an AI-sharpened map (generated using EMReady[6]), and models of the five archaellins fit into the map. Asterisks designate areas where there is a poor fit of the model in the density, with one asterisk marking density that the model cannot occupy, and two asterisks marking areas where the model is found outside the density. In regions 105-108 and 145-161 (colored orange and pink, respectively), maps of two different subunits (chains) in the filament are displayed to show the differences in density between subunits and their ability to accommodate the various models. In the region 145-161, the map corresponding to the sequence with a grey background is displayed at the lower part of the box, within the grey frame. Although some archaellin models fit the density better than others in certain regions, none of the models fit the density well in all regions, leading to ambiguity in determining the identity of the archaellins in each position within the filament. Thus, we built a consensus model for all archaellins in the filament (Supplementary Table 2).

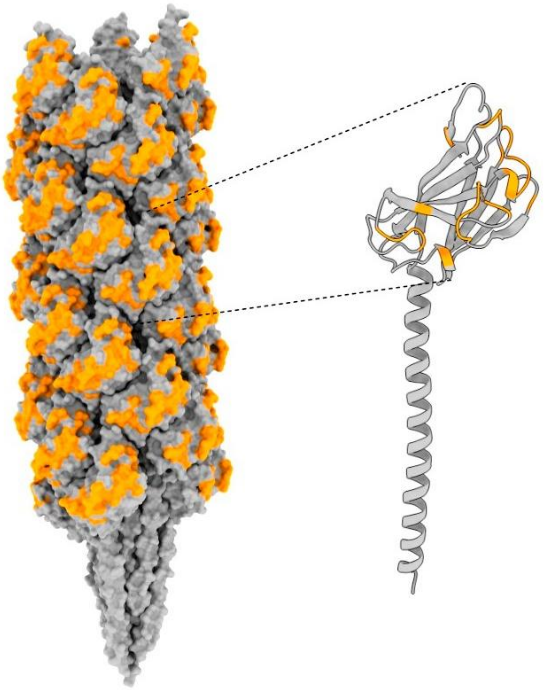

Supplementary Fig. 5. Residues that differ among archaellins face outwards. Surface presentation of a model of the *Hbt. salinarum* archaellum filament (left) and cartoon presentation of one subunit (right). Positions of amino acids that vary among the five archaellins (and which were modelled as alanine residues) are colored in orange, with how they cluster at the surface of the filament being shown. Residues that participate in inter-subunit interactions are conserved among *Hbt. salinarum* archaellins (grey).

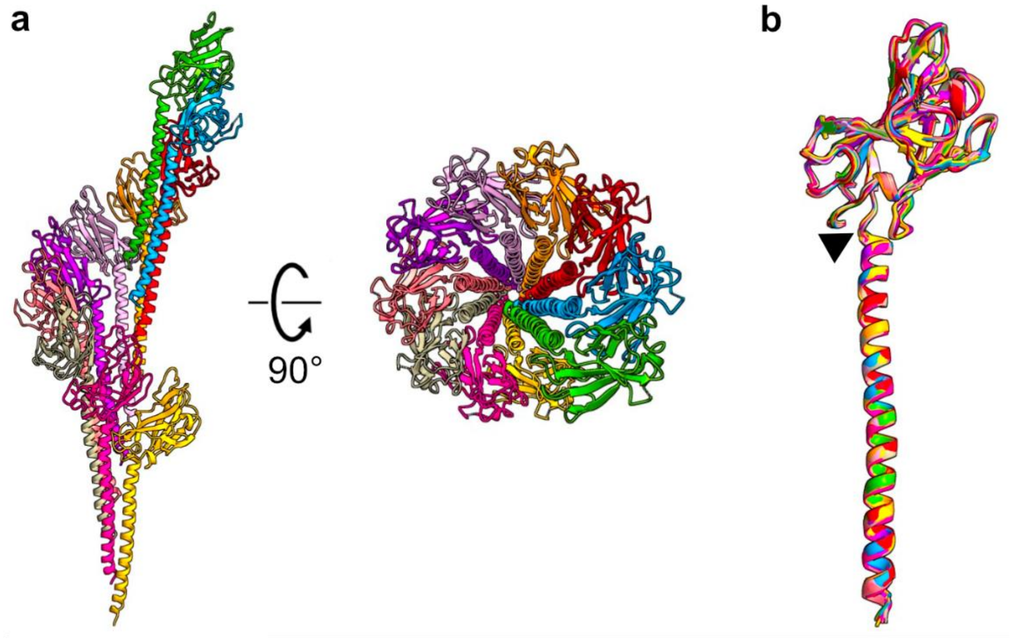

Supplementary Fig. 6. Comparisons of the conformations of archaellin subunits. a. Ten neighboring archaellins, which form one full turn within a left-handed helical strand, are shown at their positions within the archaellum filament. **b.** Alignment of the ten subunits shown in **a** display no major differences in their conformation, as opposed to the structure of the *S. islandicus* REY15A archaellum filament, which assumed a super-coiled state[7,8]. The arrowhead points to the linker region, which fuses the two archaellin domains and appears to adopt the same configuration in all subunits.

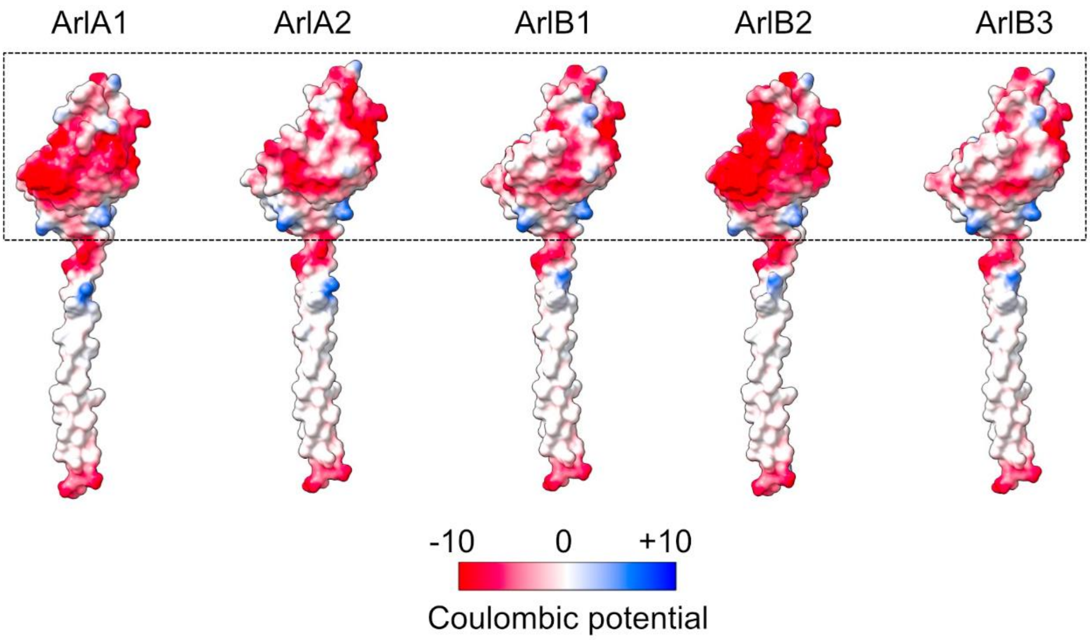

Supplementary Fig. 7. The surface of the *Hbt. salinarum* archaellum is negatively charged. Prediction models generated by AlphaFold2[5] for the five *Hbt. salinarum* archaellins. The models are colored according to Coulombic electrostatic potential, showing that the globular domains, which form the surface of the filament (indicated with a dashed box) harbor negative potential, or patches of negative potential. ArlA2, ArlB1, and ArlB3 also exhibit hydrophobic patches on their surfaces. Predictions were used here instead of our consensus model because in the latter, there are several surface residues that were modelled as alanine residues instead of charged amino acids (Supplementary Table 2), leading to under-estimation of the true electrostatic potential.

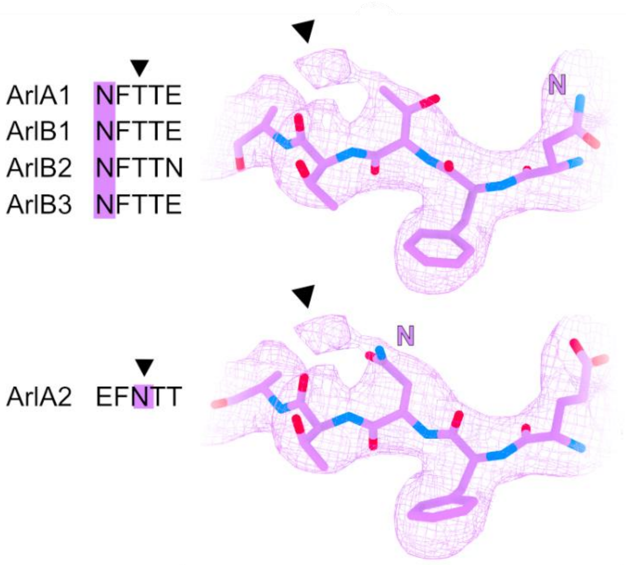

Supplementary Fig. 8. Density observed near the position of the ArlA2 residue comparable to N123 in the other archaellins. Top – cryo-EM map (shown as a mesh) and model of the sequence found in ArlA1, ArlB1, ArlB2, and ArlB3. The position of the glycosylated asparagine (N123, according to ArlB1 numbering) is indicated in the model as 'N' and in the sequence alignment (left) against a purple background. A dead-end protrusion that emerges from the threonine two positions downstream is indicated with an arrowhead. Bottom – the same cryo-EM map shown above with a model of the sequence found in ArlA2. The position of the glycosylated asparagine is shifted, relative to the other four archaellins, and is indicated in the model as 'N' and in the sequence alignment (left) against a purple background. The density observed near this asparagine is indicated with an arrowhead and presumably corresponds to some of the tetrasaccharide linked to this residue in ArlA2. This residual density was observed in only some of the subunits, suggesting that various archaellins comprise the archaellum filament, including ArlA2, such that regions that vary among archaellins produce a signal that is averaged in the cryo-EM map and appears weaker (as at this N-glycosylation site), as compared to signals from conserved regions.

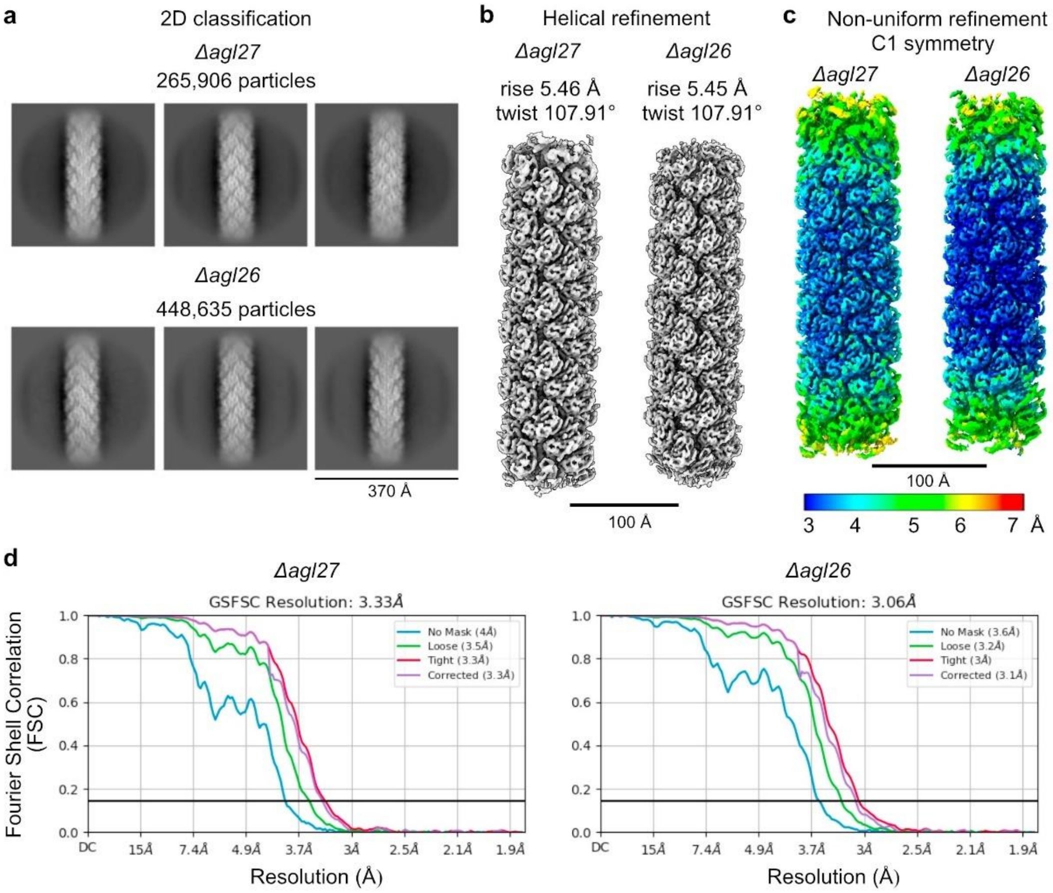

Supplementary Fig. 9. Workflow for cryo-EM data processing of archaellum filaments from the Δ*agl27* and Δ*agl26* strains. a. Representative two-dimensional class averages selected for further processing (out of 50). **b.** Cryo-EM maps of the archaellum filaments from the Δ*agl27* and Δ*agl26* strains produced by applying helical symmetry. **c.** Cryo-EM maps of the archaellum filaments from the Δ*agl27* and Δ*agl26* strains obtained without applying helical symmetry. The maps are colored according to local resolution, as estimated by cryoSPARC[1]. **d.** Fourier Shell Coefficient (FSC) measured by the Gold-standard method.

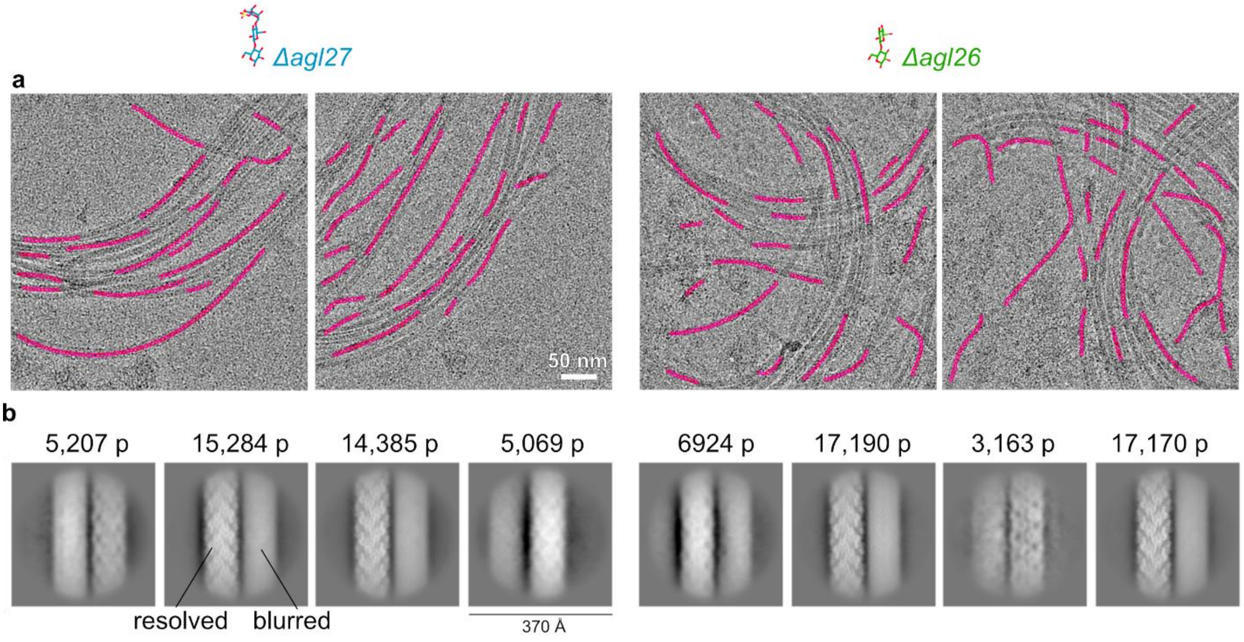

Supplementary Fig. 10. Cryo-EM micrographs of archaellum filaments from the Δ*agl27* and Δ*agl26* strains. a. Representative cryo-EM micrographs, with examples of particles picked using Filament tracer in cryoSPARC shown as pink circles[1]. Despite the appearance of bundles, we detected enough individual filaments from which to pick particles and generate cryo-EM reconstructions (Fig. 3 and Supplementary Fig. 9). For each sample, micrographs were recorded from five grids, prepared from three independent samples. **b.** 2D class averages obtained after extracting particles that contain two neighboring filaments from bundled regions. Numbers of averaged particles in each class are indicated above. As indicated for one of the classes, one filament was well-resolved, whereas the other filament was blurred.

Supplementary Tables

Supplementary Table 1 – Cryo-EM data collection, refinement, and validation statistics

Sample	Parent strain (EMDB- 19905) (PDB 9EQ7)	Δ*agl27* (EMDB-19962) (PDB 9ETU)	Δ*agl26* (EMDB-19943) (PDB 9ESM)
**Data collection and processing**			
Magnification	x130,000		
Voltage (kV)	200		
Electron exposure (e-/Å^2)	40		
Defocus range (μm)	-1.5 to -0.5		
Pixel size (Å)	0.89		
Symmetry imposed	Initial: helical (rise 5.4 Å, twist 108°); Final: C1		
Initial particle images (no.)	455,539	1,045,640	1,333,166
Final particle images (no.)	334,202	265,906	448,635
Map resolution (Å) FSC threshold 0.143	3.2	3.3	3.1
Map resolution range (Å)	3-5	3-5	3-5
**Refinement**			
Initial model used (PDB code)	AlphaFold2 prediction of AlrB1	9EQ7	9EQ7
Model resolution (Å) FSC threshold 0.5*	3.5	3.6	3.3
**Model Composition**			
Non-hydrogen atoms	36,008	33,665	32,749
Protein residues	4,664	4,453	4,431
Ligands (glycans)	52	50	50
Number of monomers	26	25	25
**RMS deviations**			
Bond length (Å)	0.003	0.002	0.002
Bond angle (°)	0.784	0.700	0.571
**Validation**			
Poor rotamers (%)	0.31	0.26	0.33
Molprobity score	1.48	1.50	1.43
Clash score	8.92	9.54	7.83
**Ramachandran Plot**			
Favored (%)	98.11	98.06	98.67
Allowed (%)	1.89	1.94	1.33
Disallowed (%)	0.00	0.00	0.00

*Models were refined against cryo-EM maps sharpened by B-factor and not AI-based methods

Supplementary Table 2 – Modelling positions that vary in sequence among archaellins

Residue number[1]	ArlA1	ArlA2	ArlB1	ArlB2	ArlB3	Consensus Model
42	F	F	Y	Y	Y	Y
72-75	KTASGTDT	NNEE	NNEK	DTSGSTEV	NSEK	UUUU[2]
77	D	D	D	N	D	D
79	A	V	V	A	V	V
95	S	S	T	S	T	U
106	T	K	R	T	K	U
112	Y	H	Y	Y	Y	Y
113-121	DGSTDADA	ANAADKTTLG	SSNSPSSLG	DGTTADA	SSNSPSSLG	UUUUUUUUU[3]
123	N	E	N	N	N	N
125	T	N	T	T	T	T
127	E	T	E	N	E	U
132-135	NNAD	NNDN	SSAD	DNAD	NNAD	UUUU
139	E	Q	D	D	E	D
145-147	KIV	KVI	KVI	EIV	KVI	KVI
149	D	Y	Y	D	Y	Y
151-156	ASSITNG	GGVSSK	SGVSSN	AEITTNG	SGVSST	UUVUUU
158-159	KA	GA	GA	KA	GS	UU
161	E	D	D	E	E	U
180	N	N	Q	N	H	Q
192	T	K	T	T	K	U

[1]Numbering is according to ArlB1
[2]U=UNK (unknown), modelled as alanine
[3]The density was too weak to trace the entire main chain in this region. Depending on the density of each chain, some of the residues were not modelled at all

Supplementary Table 3 – Measures of swimming motility

Measure	Parent strain	Δ*agl27*	Δ*agl26*
Average speed ± s.d.* (μm/sec)	2.9±0.7	2.2±0.9	2.6±0.7
Average confinement ratio ± SEM#	0.30±0.02	0.14±0.01	0.17±0.01
Average maximum distance ± SEM# (μm)	5.9±0.4	3.0±0.1	3.4±0.3

*s. d. – standard deviation
#SEM – standard error of the mean

Supplementary References

1. Punjani, A., Rubinstein, J. L., Fleet, D. J. & Brubaker, M. A. cryoSPARC: algorithms for rapid unsupervised cryo-EM structure determination. *Nat. Methods* **14**, 290–296 (2017).
2. Sievers, F. & Higgins, D. G. Clustal omega. *Curr. Protoc. Bioinforma.* **48**, 3.13.1-3.13.16 (2014).
3. Zaretsky, M., Darnell, C. L., Schmid, A. K. & Eichler, J. N-Glycosylation Is Important for Halobacterium salinarum Archaellin Expression, Archaellum Assembly and Cell Motility. *Front. Microbiol.* **10**, 1367 (2019).
4. Szabó, Z., Stahl, A. O., Albers, S. V, Kissinger, J. C., Driessen, A. J. M. & Pohlschröder, M. Identification of diverse archaeal proteins with class III signal peptides cleaved by distinct archaeal prepilin peptidases. *J. Bacteriol.* **189**, 772–778 (2007).
5. Jumper, J., Evans, R., Pritzel, A., Green, T., Figurnov, M., Ronneberger, O., Tunyasuvunakool, K., Bates, R., Žídek, A., Potapenko, A., Bridgland, A., Meyer, C., Kohl, S. A. A., Ballard, A. J., Cowie, A., Romera-Paredes, B., Nikolov, S., Jain, R., Adler, J., Back, T., Petersen, S., Reiman, D., Clancy, E., Zielinski, M., Steinegger, M., Pacholska, M., Berghammer, T., Bodenstein, S., Silver, D., Vinyals, O., Senior, A. W., Kavukcuoglu, K., Kohli, P. & Hassabis, D. Highly accurate protein structure prediction with AlphaFold. *Nature* **596**, 583–589 (2021).
6. He, J., Li, T. & Huang, S.-Y. Improvement of cryo-EM maps by simultaneous local and non-local deep learning. *Nat. Commun.* **14**, 3217 (2023).
7. Kreutzberger, M. A. B., Sonani, R. R., Liu, J., Chatterjee, S., Wang, F., Sebastian, A. L., Biswas, P., Ewing, C., Zheng, W., Poly, F., Frankel, G., Luisi, B. F., Calladine, C. R., Krupovic, M., Scharf, B. E. & Egelman, E. H. Convergent evolution in the supercoiling of prokaryotic flagellar filaments. *Cell* **185**, 3487-3500.e14 (2022).
8. Kreutzberger, M. A. B., Cvirkaite-Krupovic, V., Liu, Y., Baquero, D. P., Liu, J., Sonani, R. R., Calladine, C. R., Wang, F., Krupovic, M. & Egelman, E. H. The evolution of archaeal flagellar filaments. *Proc. Natl. Acad. Sci. U. S. A.* **120**, e2304256120 (2023).
