# Supplementary material for: Perturbed N-glycosylation of Halobacterium salinarum archaellum filaments leads to filament bundling and compromised cell motility
Source: Nat Commun. 2024 Jul 11;15:5841. doi: 10.1038/s41467-024-50277-1 (PMC11239922; doi:10.1038/s41467-024-50277-1)
Supplement: Supplementary file 3 — Description of Additional Supplementary Files [file 41467_2024_50277_MOESM3_ESM.pdf]

Description of Additional Supplementary Files:

Supplementary Movie 1: Swimming of parent, Δagl27, and Δagl26 strain cells recorded at 12.5 frames per second. Arrowheads in the Δagl27 and Δagl26 panels indicate vibrating cell clusters, which were not considered in the analyses of swimming speeds and confinement ratios.
