# Supplementary material for: Perturbed N-glycosylation of Halobacterium salinarum archaellum filaments leads to filament bundling and compromised cell motility
Source: Nat Commun. 2024 Jul 11;15:5841. doi: 10.1038/s41467-024-50277-1 (PMC11239922; doi:10.1038/s41467-024-50277-1)
Supplement: Supplementary file 5 — Reporting Summary [file 41467_2024_50277_MOESM5_ESM.pdf]

nature portfolio

Corresponding author(s): Iris Grossman-Haham

Last updated by author(s): Jun 13, 2024

Reporting Summary

Nature Portfolio wishes to improve the reproducibility of the work that we publish. This form provides structure for consistency and transparency in reporting. For further information on Nature Portfolio policies, see our Editorial Policies and the Editorial Policy Checklist.

Statistics

For all statistical analyses, confirm that the following items are present in the figure legend, table legend, main text, or Methods section.

n/a	Confirmed	
☐	☒	The exact sample size (n) for each experimental group/condition, given as a discrete number and unit of measurement
☐	☒	A statement on whether measurements were taken from distinct samples or whether the same sample was measured repeatedly
☐	☒	The statistical test(s) used AND whether they are one- or two-sided<br>*Only common tests should be described solely by name; describe more complex techniques in the Methods section.*
☒	☐	A description of all covariates tested
☒	☐	A description of any assumptions or corrections, such as tests of normality and adjustment for multiple comparisons
☐	☒	A full description of the statistical parameters including central tendency (e.g. means) or other basic estimates (e.g. regression coefficient) AND variation (e.g. standard deviation) or associated estimates of uncertainty (e.g. confidence intervals)
☐	☒	For null hypothesis testing, the test statistic (e.g. F, t, r) with confidence intervals, effect sizes, degrees of freedom and P value noted<br>*Give P values as exact values whenever suitable.*
☒	☐	For Bayesian analysis, information on the choice of priors and Markov chain Monte Carlo settings
☒	☐	For hierarchical and complex designs, identification of the appropriate level for tests and full reporting of outcomes
☒	☐	Estimates of effect sizes (e.g. Cohen's d, Pearson's r), indicating how they were calculated

Our web collection on statistics for biologists contains articles on many of the points above.

Software and code

Policy information about availability of computer code

Data collection	cryo-EM data collection: EPU (Thermo Fisher Scientific) v3.4.0, negative-stain TEM: Velox (Thermo Fisher Scientific) v3.12.0, light microscopy: SlideBook
Data analysis	cryo-EM data processing: cryoSPARC (v4.3.0), DeepEMhancer, EMready v2.0; model building: ccp4 v8.0, COOT v0.9.8.8, PHENIX v1.21rc1-4985-000; structure analysis: UCSF ChimeraX 1.8, D, PyMol v2.5.7; swimming analyses: ImageJ v2.14.0, TrackMate v7.11.1; statistical analyses: Prism v10.2.2 (GraphPad).

For manuscripts utilizing custom algorithms or software that are central to the research but not yet described in published literature, software must be made available to editors and reviewers. We strongly encourage code deposition in a community repository (e.g. GitHub). See the Nature Portfolio guidelines for submitting code & software for further information.

Data

Policy information about availability of data

All manuscripts must include a data availability statement. This statement should provide the following information, where applicable:

- Accession codes, unique identifiers, or web links for publicly available datasets
- A description of any restrictions on data availability
- For clinical datasets or third party data, please ensure that the statement adheres to our policy

Source data are provided with this paper. The atomic coordinates generated in this study for the Hbt. salinarum archaellum filament from the parent, Δagl27, and Δagl26 strains have been deposited in the Protein Data Bank (https://www.rcsb.org/) under accession numbers 9EQ7, 9ETU, and 9ESM, respectively. The electron

density maps for the archaellum filaments from parent, Δagl27, and Δagl26 Hbt. salinarum strain have been deposited in the EM DataResource (https://www.emdataresource.org/) under the accession numbers EMDB–19905, EMDB–19962, and EMDB–19943, respectively.

Research involving human participants, their data, or biological material

Policy information about studies with human participants or human data. See also policy information about sex, gender (identity/presentation), and sexual orientation and race, ethnicity and racism.

Reporting on sex and gender	N/A
Reporting on race, ethnicity, or other socially relevant groupings	N/A
Population characteristics	N/A
Recruitment	N/A
Ethics oversight	N/A

Note that full information on the approval of the study protocol must also be provided in the manuscript.

Field-specific reporting

Please select the one below that is the best fit for your research. If you are not sure, read the appropriate sections before making your selection.

☒ Life sciences ☐ Behavioural & social sciences ☐ Ecological, evolutionary & environmental sciences

For a reference copy of the document with all sections, see nature.com/documents/nr-reporting-summary-flat.pdf

Life sciences study design

All studies must disclose on these points even when the disclosure is negative.

Sample size	Sample sizes were not predetermined, as the effect of sample size were unknown. The same number of movies was recorded for each sample (fig.5) and all cells in these movies were analysed, yielding similar but not identical sample sizes. For micrographs of negatively-stained cells, all regions in the grid that had proper stain were imaged.
Data exclusions	No data was excluded for whole cell imaging, every cell that was imaged was included in the sample (both in TEM and motility experiments).
Replication	For every strain, negative-stain imaging was performed on at least three cultures grown independently and motility imaging was performed on at least two independent cultures, which were recorded in at least three independent movies. All attempts at replication are reported and were successful.
Randomization	Randomization was not applied in this study. Samples were organized into groups based on the strain they came from (parent, Δagl27, or Δagl26).
Blinding	Blinding was not performed; Blinding was not possible since we noted the bundling effect of filaments while looking at the grids for the first time.

Reporting for specific materials, systems and methods

We require information from authors about some types of materials, experimental systems and methods used in many studies. Here, indicate whether each material, system or method listed is relevant to your study. If you are not sure if a list item applies to your research, read the appropriate section before selecting a response.

Materials & experimental systems

n/a	Involved in the study	
☒	☐	Antibodies
☒	☐	Eukaryotic cell lines
☒	☐	Palaeontology and archaeology
☒	☐	Animals and other organisms
☒	☐	Clinical data
☒	☐	Dual use research of concern
☒	☐	Plants

Methods

n/a	Involved in the study	
☒	☐	ChIP-seq
☒	☐	Flow cytometry
☒	☐	MRI-based neuroimaging

Plants

Seed stocks	N/A
Novel plant genotypes	N/A
Authentication	N/A
